# Recorde de Resistência em 366 Maratonas durante 366 Dias: Um Estudo de Caso

**DOI:** 10.36660/abc.20240838

**Published:** 2025-04-29

**Authors:** Francis Ribeiro de Souza, Renato Delascio Lopes, Guilherme Wesley Peixoto da Fonseca, Rodrigo Bellio de Mattos Barretto, Antonio Carlos Battaglia, Renata Margarida do Val, Roberto Kalil-Filho, Maria-Janieire de Nazaré Nunes Alves

**Affiliations:** 1 Hospital das Clínicas Faculdade de Medicina Universidade de São Paulo São Paulo SP Brasil Instituto do Coração do Hospital das Clínicas da Faculdade de Medicina da Universidade de São Paulo, São Paulo, SP – Brasil; 2 Duke University Hospital Durham North Carolina EUA Duke University Hospital, Durham, North Carolina – EUA; 3 Escola de Educação Física e Esporte Universidade de São Paulo São Paulo SP Brasil Escola de Educação Física e Esporte da Universidade de São Paulo, São Paulo, SP – Brasil; 4 Hospital Sírio Libanês São Paulo SP Brasil Hospital Sírio Libanês, São Paulo, SP – Brasil

**Keywords:** Corrida de Maratona, Sistema Cardiovascular, Resistência Física

## Abstract

**Fundamento:**

Um atleta brasileiro propôs estabelecer um novo recorde mundial de maratonas consecutivas, competindo em 366 maratonas durante 366 dias consecutivos. O impacto dessas maratonas consecutivas sobre o sistema cardiovascular permanece desconhecido.

**Objetivo:**

Monitorar o sistema cardiovascular para avaliar quaisquer adaptações cardiovasculares do atleta ao longo do período.

**Métodos:**

Durante a avaliação pré-estudo, conduzimos uma avaliação clínica pré-participação (APP) composta por anamnese, eletrocardiograma, exame de sangue e capacidade funcional por meio do teste cardiopulmonar no exercício (TCPE). Durante o acompanhamento, o TCPE seriado, a avaliação da composição corporal, a análise de amostra de sangue e o ecocardiograma foram realizados periodicamente, por 12 meses.

**Resultados:**

Na APP, indivíduo do sexo masculino, 43 anos, altura: 1,83 m, peso: 76,9 kg, consumo máximo de oxigênio (VO_2_máx): 52 ml/kg/min, gordura corporal: 12,6%, pressão arterial sistólica e diastólica: 120/80 mmHg, glicemia: 92 mg/dL, colesterol total: 185 mg/dL, proteína C reativa de alta sensibilidade (hs-CRP): 0,08 mg/dL, creatina fosfoquinase (CPK): 183 U/L e troponina T de alta sensibilidade (hs-TnT): 7,1 ng/L. Durante o acompanhamento, a média de VO_2_máx permaneceu em 48,7 ± 1,2 ml/kg/min, a Fração de Ejeção do Ventrículo Esquerdo (FEVE) em 62 ± 2%, o strain longitudinal global do VE em 19 ± 1%, o índice de massa do VE em 83 ± 7 g/m^2^, o hs-CRP em 0,07 ± 0,01 mg/L, a CPK em 169 ± 36 U/L, a hs-TnT em 8,2 ± 1,4 ng/L e nenhuma arritmia maligna foi observada.

**Conclusão:**

O sistema cardiovascular do atleta se adaptou a um volume extremamente alto de maratonas consecutivas em intensidade moderada por 1 ano e permaneceu funcional na faixa de normalidade. Além disso, o atleta estabeleceu um novo recorde mundial de maratonas consecutivas, reconhecido pelo Guinness World Records.

## Graphical abstract

**Figura Central:**
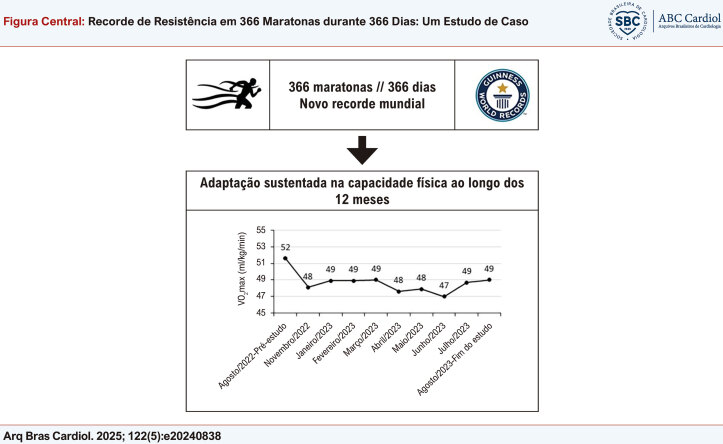
Recorde de Resistência em 366 Maratonas durante 366 Dias: Um Estudo de CasoIntrodução

## Introdução

O desempenho de resistência é determinado pelo consumo máximo de oxigênio (VO_2_máx), economia de corrida e limiar de lactato.^1^ Por meio do treinamento físico regular, é possível desenvolver a economia de corrida, especialmente em corredores de longa distância.^2^ Robison e colaboradores relataram o perfil fisiológico de um maratonista do sexo masculino, de 70 anos de idade, que correu uma maratona em 2:54:23 em 15 de dezembro de 2018, quebrando o tempo recorde mundial para homens com mais de 70 anos.^3^

Um atleta belga correu uma maratona por dia durante 365 dias em 2010, realizando um dos maiores feitos da história do esporte.^4^ No entanto, nenhuma avaliação clínica foi realizada e, portanto, não existem informações disponíveis sobre o impacto das maratonas diárias sobre o sistema cardiovascular.

Um maratonista amador brasileiro propôs estabelecer um novo recorde mundial de maratonas consecutivas, correndo 366 maratonas em 366 dias consecutivos. O impacto dessas maratonas consecutivas sobre o sistema cardiovascular permanece desconhecido.

O objetivo deste estudo foi monitorar o sistema cardiovascular regularmente para avaliar quaisquer adaptações cardiovasculares do atleta ao longo do período de 366 maratonas consecutivas.

## Materiais e métodos

### População do estudo

O comitê local para a Proteção de Seres Humanos aprovou este estudo (CAAE: 61097822.0.0000.0068). Avaliamos um maratonista brasileiro (H.L.S.F), que correu 366 maratonas consecutivas em 366 dias (de 28 de agosto de 2022 a 28 de agosto de 2023). O participante deu seu consentimento informado por escrito.

### Avaliação pré-estudo

A avaliação clínica pré-participação (APP) foi realizada de acordo com a Sociedade Brasileira de Medicina do Exercício e do Esporte.^5^ Realizamos uma anamnese e um exame clínico composto por eletrocardiograma (ECG) de repouso de 12 derivações, teste cardiopulmonar de exercício máximo (TCPE), análise de bioimpedância elétrica (ABI) e exame de sangue Além disso, o atleta apresentou um ecocardiograma (ECO) na APP, realizada três meses antes do nosso estudo.

### Acompanhamento

Todos os testes foram repetidos periodicamente. No terceiro mês de acompanhamento, incluímos também medidas de estrutura cardíaca, funções dos ventrículos esquerdo (VE) e direito (VD) por ECO, repetindo-as até o final do estudo.

Além disso, uma equipe multiprofissional composta por dermatologista, endocrinologista, ortopedista, fisioterapeuta, nutricionista, preparador físico e psicólogo ofereceu suporte ao atleta durante o período do estudo.

### Teste Cardiopulmonar de Exercício (TCPE)

O TCPE foi realizado utilizando o esforço máximo em esteira automática (Embramed – Modelo – Atlanta, Estados Unidos). Um analisador de gases respiratórios computadorizado (Vynthus CPX – Pulmonary Function/Cardiopulmonary Exercise Testing Instrument, Hoechberg, Alemanha) foi utilizado para avaliar a ventilação pulmonar, o consumo máximo de oxigênio (VO_2_máx) e a produção de dióxido de carbono (VCO_2_). As variáveis foram medidas a cada respiração. O primeiro limiar anaeróbio (LA) foi identificado pela técnica “V-slope”, observado no primeiro ponto de dissociação das curvas VE/VO_2_ e no menor valor da pressão parcial de oxigênio ao final da expiração (PetO_2_), antes que esse parâmetro começasse a aumentar progressivamente. O segundo limiar ventilatório (ponto de compensação respiratória) foi identificado pela inflexão nas curvas VE/VCO_2_ e o valor máximo de pressão parcial para CO_2_ ao final da expiração (PetCO_2_), antes de uma diminuição progressiva dessa resposta.^6^ A frequência cardíaca (FC) foi registrada continuamente durante o TCPE, usando um ECG de 12 derivações e o software CardioSoft v6. O TCPE foi realizado na APP, após três meses, e mensalmente até o final do estudo. O atleta correu as maratonas pela manhã e os TCPEs foram realizados à tarde.

### Ecocardiograma

As imagens foram coletadas pelo Vivid E9 (GE Healthcare; Oslo, Noruega). O atleta foi submetido a um ECO bidimensional. O Doppler colorido de 4 câmaras e, em seguida, o Doppler de onda de pulso foram usados para avaliar as velocidades de pico de fluxo através da válvula mitral. Avaliamos as vistas paraesternais do eixo longo, a espessura da parede septal e posterior do VE na diástole, bem como as dimensões da câmara do VE ao final da diástole e ao final da sístole. O volume do VE ao final da diástole e o volume do VE ao final da sístole foram avaliados por meio de vistas apicais de 2 e 4 câmaras, que permitiram a estimativa do volume sistólico e da fração de ejeção do ventrículo esquerdo (FEVE) pelo método biplano de Simpson. O software específico de rastreamento de marcas (speckle-tracking) foi usado para estimar o strain em todos os segmentos. Todas as imagens foram coletadas de acordo com a Sociedade Americana de Ecocardiografia e a Associação Europeia de Imagem Cardiovascular.^7^ O primeiro ECO foi realizado três meses após o início do estudo e avaliado periodicamente durante o acompanhamento. O atleta correu as maratonas pela manhã e os ECOs foram realizados à tarde, sempre antes do TCPE.

### Análise estatística

Os dados são apresentados periodicamente com base em avaliações e relatados como média ± desvio padrão (DP). O Statistical Package for Social Science (SPSS) versão 23 foi usado para descrever as variáveis.

## Resultados

### Avaliação pré-estudo

Na APP, indivíduo de 43 anos de idade, altura: 1,83 m, peso: 76,9 kg, VO_2_max: 52 ml/kg/min, gordura corporal: 12,6%, pressão arterial sistólica e diastólica: 120/80 mmHg, glicemia: 92 mg/dL, colesterol total: 185 mg/dL, proteína C reativa de alta sensibilidade (hs-CRP): 0,08 mg/dL, creatina fosfoquinase (CPK): 183 U/L e troponina T de alta sensibilidade (hs-TnT): 7,1 ng/L.

No primeiro TCPE, utilizamos um protocolo de rampa específico (velocidade 1) composto por incrementos de velocidade e inclinação a cada minuto até a exaustão do atleta (detalhes do protocolo são descritos em material complementar). A duração do TCPE foi de 10 minutos, com velocidade máxima de 8,2 mph, 11,0% de inclinação, VO_2_máx de 52,0 ml/kg/min e razão de troca respiratória (R) de 1,11. Um R ≥ 1,10 é considerada como teste máximo.^6^ O primeiro LA estava em torno de 6,0 mph e a FC foi de 150 bpm. O atleta foi instruído a correr as maratonas em intensidade moderada, próxima ao primeiro limiar anaeróbico (LA) obtido pelo TCPE máximo.

### Acompanhamento

As medidas clínicas, a composição corporal, os biomarcadores e o perfil lipídico são descritos na Tabela 1. Essas variáveis apresentaram variações fisiológicas mínimas, sem alterações anormais. A FC de repouso foi medida a partir do TCPE em posição supina após 2 minutos em repouso. A ligeira variação da FC de repouso pode estar relacionada à ansiedade pré-teste.

**Tabela 1:** – Medidas clínicas, composição corporal, biomarcadores e perfil lipídico

Variáveis	Agosto de 2022, Pré-estudo	2022 Novembro	2023 Fevereiro	2023 Maio	2023 Julho	Agosto de 2023, Fim do estudo	Média - DP	Valor normal ou intervalo
**Clínico**								
PAS (mmHg)	120	110	110	110	120	120	115 ± 5	< 120
PAD (mmHg)	90	80	80	80	80	80	80 ± 5	< 80
FC em repouso (bpm)	90	81	96	80	67	77	82 ± 10	< 100
**Composição corporal**								
Peso (kg)	76,9	74,2	71,4	71,0	71,7	70,5	72,6 ± 2,5	-
Altura (m)	1,83	1,83	1,83	1,83	1,83	1,83	1,83	-
IMC (Kg/m^2^)	23,0	22,2	21,3	21,2	21,4	21,1	21,7 ± 0,7	18,9 - 24,9
Massa magra (kg)	67,2	65,5	64,2	63,9	64,7	63,6	64,9 ± 1,3	-
Massa gorda (kg)	9,7	8,7	7,2	7,1	7,0	6,9	7,8 ± 1,2	-
Massa gorda (%)	12,6	11,7	10,1	10,0	9,7	9,8	10,7 ± 1,2	< 20,0
**Biomarcadores e perfil lipídico**								
hs-CRP (mg/L)	0,08	0,08	0,06	0,08	0,07	0,06	0,07 ± 0,01	< 0,1
CPK (U/L)	183	184	190	190	183	104	169 ± 36	46 - 171
hs-TnT (ng/L)	7,1	6,0	8,8	10,0	8,0	9,0	8,2 ± 1,4	< 16,8
Creatinina (mg/dL)	1,14	1,01	1,15	1,02	0,99	1,06	1,06 ± 0,07	0,7 - 1,30
Potássio (mmol/L)	4,8	4,3	4,5	4,3	4,7	4,5	4,5 ± 0,2	3,5 - 5,1
Sódio (mmol/L)	137	136	141	138	139	140	139 ± 2	136 - 145
NT-proBNP (pg/mL)	-	-	22,1	24,4	10,8	14,4	17,9 ± 6,4	< 125
CT (mg/dL)	185	180	-	199	191	204	192 ± 10	< 190
LDL (mg/dL)	111	111	-	130	126	132	122 ± 10	< 130
HDL (mg/dL)	53	48	-	52	49	56	52 ± 3	> 40
TG (mg/dL)	99	81	-	75	64	78	79 ± 13	< 150

*FC: frequência cardíaca; PAS: pressão arterial sistólica; PAD: pressão arterial diastólica; IMC: índice de massa corporal; hs-CRP: proteína C-reativa de alta sensibilidade; CPK: creatina fosfoquinase; hs-TnT: troponina T de alta sensibilidade; NT-ProBNP: proBNP N-terminal; LDL: lipoproteína de baixa densidade; HDL: lipoproteína de alta densidade; TG: triglicerídeos; CT: colesterol total.*

**Figura 1:**
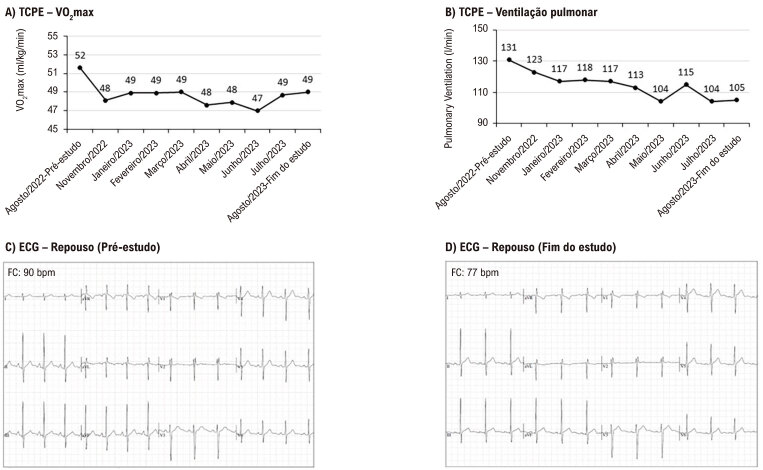
– Acompanhamento do consumo máximo de oxigênio (VO2max) e ventilação pulmonar por teste de exercício cardiopulmonar (TCPE), (Fig. 1 A-B, respectivamente). Eletrocardiograma de repouso (ECG) de 12 derivações no pré-estudo e ECG ao final do estudo (Fig. 1B-C, respectivamente).

A FC média durante as maratonas foi de 140 ± 10 bpm, o VO_2_máx permaneceu em 49 ± 1 ml/kg/min e a ventilação pulmonar em 113 ± 4 L/min (Figura 1 A-B). A partir do TCPE realizado em janeiro, a velocidade do protocolo 1 foi alterada para o protocolo D, a fim de evitar o risco de lesão muscular durante o TCPE (material complementar). Essa alteração pode justificar a ligeira queda no VO_2_máx e na ventilação. No entanto, o atleta atingiu a FC máxima (FC > 95% prevista pela idade), com R de 1,08 ± 0,04. Além disso, não foram encontrados sinais de arritmia maligna e distúrbios cardiovasculares durante o TCPE, o que nos garantiu a segurança de prosseguir com as maratonas. Alterações significativas entre o ECG de 12 derivações no pré-estudo e o ECG de 12 derivações no final do estudo também não foram identificadas (Figura 1B-C).

As variáveis estruturais cardíacas, a função sistólica do VE, a função sistólica do VD e o trabalho miocárdico são descritas na Tabela 2. Essas variáveis apresentaram variações fisiológicas mínimas dentro dos valores normais.

**Tabela 2:** – Estrutura cardíaca, função sistólica do ventrículo esquerdo, função sistólica do ventrículo direito e trabalho miocárdico

Variáveis	Outubro Após 3 meses	2022 Novembro	2023 Março	2023 Junho	2023 Julho	Agosto de 2023 Fim do estudo	Média - DP	Valor normal ou intervalo
**Estruturas cardíacas**								
Raiz aórtica (cm)	3,4	3,3	3,4	3,3	3,3	3,4	3,4 ± 0,1	3,0 - 3,7
Diâmetro do AD (cm)	3,8	3,5	3,6	3,3	3,4	3,3	3,5 ± 0,1	3,0 - 4,0
VD basal (cm)	3,8	3,8	3,4	4,0	4,0	3,3	3,7 ± 0,3	2,5 - 4,1
VD médio (cm)	3,1	3,1	2,8	3,1	3,3	2,7	3,0 ± 0,2	1,9 - 3,5
Septo interventricular (cm)	0,9	0,8	0,9	0,8	0,9	0,8	0,9 ± 0,1	0,6 - 1,0
Espessura da parede posterior do VE (cm)	0,9	0,8	0,8	0,8	0,8	0,8	0,8 ± 0,1	0,6 - 1,0
Diâmetro diastólico final do VE (cm)	5,4	5,3	5,4	5,3	5,5	5,2	5,4 ± 0,1	4,2 - 5,8
Diâmetro sistólico final do VE (cm)	3,5	3,7	3,6	3,4	3,4	3,3	3,5 ± 0,1	2,5 - 4,0
Volume diastólico do VE (ml)	157	141	153	149	152	149	150 ± 5	80-150
Volume sistólico do VE (ml)	62	63	58	59	60	62	61 ± 2	70
Índice de massa do VE (g/m^2^)	92	77	87	78	90	76	83 ± 7	49 - 115
Índice de volume do AE (ml/m^2^)	18	20	21	16	25	22	20 ± 3	< 34
**Função sistólica do ventrículo esquerdo**								
Fração de ejeção do VE (%)	60	62	62	61	60	59	61 ± 1	> 52
Strain longitudinal global do VE (%)	16	18	19	19	20	19	19 ± 2	≥ 18
**Função sistólica do ventrículo direito**								
TAPSE (mm)	21	24	24	22	24	25	23 ± 2	> 17
S’ (cm/s)	14	13	14	14	15	19	15 ± 2	> 9,5
CAF (%)	49	46	53	51	46	50	49 ± 3	> 35
Strain do VD (%)	21	24	23	25	23	22	23 ± 1	> 20
**Trabalho miocárdico**								
EFG (%)	95	-	92	92	97	94	94 ± 2	> 96
TGP (mmHg)	84	-	182	154	49	106	115 ± 53	73 - 87
IGT (mmHg%)	1576	-	1953	1688	2148	2025	1878 ± 238	1907 - 2113
TCG (mmHg%)	1680	-	2194	1987	2224	2209	2059 ± 233	2186 - 2369

*AD: átrio direito; VD: ventrículo direito; VE: ventrículo esquerdo; AE: átrio esquerdo; TAPSE: excursão sistólica do plano do anel tricúspide; S’: onda de velocidade sistólica do anel tricúspide lateral; CAF: coeficiente de alteração fracional; EFG: eficiência funcional global; TGP: trabalho global perdido; IGT: índice global de trabalho; TCG: trabalho construtivo global.*

Além disso, o atleta estava participando de um programa de fortalecimento muscular baseado em treinamento com pesos, mobilidade, flexibilidade e alongamento, duas vezes por semana, 2 a 3 séries para cada grupo muscular com 15 repetições a 70% de sua capacidade máxima durante as maratonas. O consumo médio diário de calorias foi de 4.716 ± 806 quilocalorias (composto por carboidratos 68 ± 6%, lipídios 17 ± 5% e proteína 15 ± 4%). Um trabalho psicológico também foi realizado pelo atleta, composto por 102 sessões de terapia que avaliaram aspectos pessoais, motivacionais, de resiliência, capacidade de lidar com frustrações, envolvimento familiar, divisão de rotina e adaptação ao longo do período. Em janeiro de 2023, o atleta foi diagnosticado com pubalgia e precisou de tratamento fisioterapêutico, que permaneceu até agosto de 2023. O atleta documentou todas as maratonas e distâncias usando dois smart-watches simultaneamente e utilizou 27 pares de tênis de corrida. O tempo médio da maratona foi de 5 horas, 8 minutos e 28 segundos, sendo o mais rápido de 3 horas, 54 minutos e 48 segundos e o mais lento de 10 horas 21 minutos e 6 segundos.

## Discussão

Até onde sabemos, este é o primeiro estudo a monitorar regularmente o sistema cardiovascular de forma abrangente em um maratonista que correu 366 maratonas consecutivas diárias. Nós hipotetizamos que o atleta passaria por adaptações cardiovasculares fisiológicas ou má adaptação devido ao alto volume de corrida em um ano. Interessantemente, o atleta apresentou variações fisiológicas mínimas, sem alterações cardiovasculares anormais em um ano. Além disso, o atleta estabeleceu um novo recorde mundial, correndo 366 maratonas consecutivas em 366 dias, conforme reconhecido pelo Guinness World Records.^8^

Primeiramente, realizamos a APP para identificar a presença de quaisquer doenças cardiovasculares desconhecidas que poderiam ser incompatíveis com este estudo. O atleta não tinha histórico familiar de morte cardíaca súbita, cardiomiopatia hipertrófica e apresentou ótima resposta cardiovascular, respiratória, hemodinâmica e física durante o TCPE.^9^ O ECG de repouso de 12 derivações, o exame de sangue e a pressão arterial estavam dentro dos limites normais. Além disso, o atleta apresentou um ECO com valores normais, realizado três meses antes do nosso estudo.

O treinamento físico é um gatilho poderoso para promover a remodelação estrutural e funcional do coração.^10^ No entanto, há um reconhecimento crescente do impacto do treinamento físico prolongado sobre a remodelação cardíaca, que pode eventualmente simular determinadas condições patológicas.^11^ Durante o acompanhamento, monitoramos periodicamente o atleta para garantir sua segurança e, caso ele apresentasse qualquer condição patológica que pudesse progredir de forma prejudicial, com risco potencial à sua vida, o estudo teria sido interrompido. Curiosamente, o atleta apresentou variações fisiológicas mínimas na estrutura cardíaca e na função do VE e do VD, sem sinais de overtraining ou inflamação alterada ao longo do período.^12^

De forma controversa, o treinamento físico pode estar associado ao aumento do risco de arritmias ventriculares e atriais.^13^ Além disso, a intensidade do exercício - mas não o volume - pode estar associada ao aumento da calcificação coronária em atletas.^14^ Em nosso estudo, o atleta foi instruído a correr as maratonas em intensidade moderada (próximo ao primeiro LA), e nenhum sinal de ECG alterado, sugerindo isquemia e arritmias malignas, foi observado durante o TCPE.

Estudos têm sido realizados por vários anos para entender as respostas fisiológicas de atletas com registros de desempenho.^1^ O VO_2_máx é um marcador da resposta respiratória e circulatória cardíaca, sendo um dos principais fatores que contribuem para o desempenho de resistência.^1^ Além disso, o consumo de oxigênio em estado estacionário é frequentemente chamado de economia de corrida (ou, pelo termo em inglês “running economy”). O maratonista, um homem de 70 anos, que correu uma maratona de menos de 3 horas em 2018, quebrando o recorde mundial para homens com mais de 70 anos, realizou um único TCPE e demonstrou um VO_2_máx de aproximadamente 46,9 ml/kg/min. Este valor foi excepcional para sua idade, uma vez que o VO_2_máx médio para um homem da mesma faixa etária é de cerca de 26 ml/kg/min.^3^ Em contraste, nenhuma avaliação clínica foi realizada no atleta belga que correu 365 maratonas consecutivas, destacando a singularidade e a significância de nossas descobertas.

Em nosso estudo, os TCPEs foram realizados à tarde, após o atleta ter completado uma maratona no início do dia. Esse fato pode ter influenciado as métricas de desempenho, incluindo VO_2_máx, limiares ventilatórios e respostas cardíacas, subestimando potencialmente a verdadeira capacidade máxima do atleta. No entanto, o atleta mostrou um VO_2_máx ideal, com adaptações sustentadas na capacidade física ao longo dos 12 meses (Figura Central).

### Implicação clínica

Esses resultados destacam a importância de uma avaliação cardiovascular cuidadosa em atletas submetidos a exercícios de resistência e oferecem uma visão única sobre a relação entre maratonas de intensidade moderada de alto volume e adaptações cardiovasculares.

### Limitações

Reconhecemos algumas limitações em nosso estudo. Do TCPE realizado em janeiro até agosto, ajustamos a velocidade do protocolo para reduzir o risco de lesão muscular durante o TCPE. Essa alteração pode explicar a leve queda observada no VO_2_máx e na ventilação, o que pode limitar a utilidade científica do VO_2_máx como variável de acompanhamento. No entanto, o VO_2_máx permaneceu em torno de 49 ml/kg/min, sugerindo uma capacidade funcional sustentada. O ECO não foi realizado no estudo de pré-avaliação, pois o atleta apresentou um ECO com valores normais na APP realizada três meses antes do nosso estudo. Porém, durante o acompanhamento, os parâmetros estavam dentro dos limites normais. Este estudo se refere a um maratonista do sexo masculino, e esses resultados devem ser interpretados com cautela ao extrapolar para uma população mais ampla.

## Conclusão

Em conclusão, o sistema cardiovascular do atleta se adaptou a um volume extremamente alto de maratonas consecutivas e permaneceu funcional na faixa de normalidade. Além disso, o atleta estabeleceu um novo recorde mundial, correndo 366 maratonas consecutivas em 366 dias, conforme reconhecido pelo Guinness World Records.

## References

[1] Joyner MJ, Coyle EF (2008). Endurance Exercise Performance: The Physiology of Champions. J Physiol.

[2] Barnes KR, Kilding AE (2015). Running Economy: Measurement, Norms, and Determining Factors. Sports Med Open.

[3] Robinson AT, Watso JC, Babcock MC, Joyner MJ, Farquhar WB (2019). Record-Breaking Performance in a 70-Year-Old Marathoner. N Engl J Med.

[4] British Broadcasting Corporation (2011). Belgian Stefaan Engels Completes Record 365th Marathon.

[5] Ghorayeb N, Stein R, Daher DJ, Silveira ADD, Ritt LEF, Santos DFPD (2019). The Brazilian Society of Cardiology and Brazilian Society of Exercise and Sports Medicine Updated Guidelines for Sports and Exercise Cardiology - 2019. Arq Bras Cardiol.

[6] Balady GJ, Arena R, Sietsema K, Myers J, Coke L, Fletcher GF (2010). Clinician's Guide to Cardiopulmonary Exercise Testing in Adults: A Scientific Statement from the American Heart Association. Circulation.

[7] Lang RM, Badano LP, Mor-Avi V, Afilalo J, Armstrong A, Ernande L (2015). Recommendations for Cardiac Chamber Quantification by Echocardiography in Adults: An Update from the American Society of Echocardiography and the European Association of Cardiovascular Imaging. J Am Soc Echocardiogr.

[8] Guinness World Records (2023). Most Consecutive Days to Run a Marathon Distance (Male).

[9] Herdy AH, Caixeta A (2016). Brazilian Cardiorespiratory Fitness Classification Based on Maximum Oxygen Consumption. Arq Bras Cardiol.

[10] La Gerche A, Wasfy MM, Brosnan MJ, Claessen G, Fatkin D, Heidbuchel H (2022). The Athlete's Heart-Challenges and Controversies: JACC Focus Seminar 4/4. J Am Coll Cardiol.

[11] Maron BJ, Pelliccia A (2006). The Heart of Trained Athletes: Cardiac Remodeling and the Risks of Sports, Including Sudden Death. Circulation.

[12] (2016). Recommendations for Cardiac Chamber Quantification by Echocardiography in Adults: An Update from the American Society of Echocardiography and the European Association of, Cardiovascular Imaging. Eur Heart J Cardiovasc Imaging.

[13] Heidbuchel H (2018). The Athlete's Heart is a Proarrhythmic Heart, and What That Means for Clinical Decision Making. Europace.

[14] Aengevaeren VL, Mosterd A, Bakker EA, Braber TL, Nathoe HM, Sharma S (2023). Exercise Volume versus Intensity and the Progression of Coronary Atherosclerosis in Middle-Aged and Older Athletes: Findings from the MARC-2 Study. Circulation.

