# Supplementary material for: Recorde de Resistência em 366 Maratonas durante 366 Dias: Um Estudo de Caso
Source: Arq Bras Cardiol. 2025 Apr 29;122(5):e20240838. [Article in Portuguese] doi: 10.36660/abc.20240838 (PMC12129474; doi:10.36660/abc.20240838)
Supplement: Translation

# Translation: Record-Breaking Endurance of 366 Marathons in 366 Days: A Case Study

**DOI:** 10.36660/abc.20240838i

**Authors:** Francis Ribeiro de Souza, Renato Delascio Lopes, Guilherme Wesley Peixoto da Fonseca, Rodrigo Bellio de Mattos Barretto, Antonio Carlos Battaglia, Renata Margarida do Val, Roberto Kalil-Filho, Maria-Janieire de Nazaré Nunes Alves

**Affiliations:** 1 Brasil Instituto do Coração do Hospital das Clínicas da Faculdade de Medicina da Universidade de São Paulo, São Paulo, SP – Brasil; 2 USA Duke University Hospital, Durham, North Carolina – USA; 3 Brazil Escola de Educação Física e Esporte da Universidade de São Paulo, São Paulo, SP – Brazil; 4 Brazil Hospital Sírio Libanês, São Paulo, SP – Brazil

**Keywords:** Marathon Running, Cardiovascular System, Physical Endurance

## Abstract

**Background:**

A Brazilian athlete has proposed setting a new world record for consecutive marathons by running 366 marathons in 366 consecutive days. The impact of such a feat on the cardiovascular system is unknown.

**Objective:**

To monitor the cardiovascular system to assess the athlete’s cardiovascular adaptations or maladaptations over the period.

**Methods:**

During the pre-study evaluation, we conducted the pre-participation clinical evaluation (PPE) composed of anamnesis, electrocardiogram, blood test, and functional capacity by maximum cardiopulmonary exercise test (CPET). At follow-up, serial CPET, body composition assessment, blood sample, and echocardiogram were periodically performed for 12 months.

**Results:**

At PPE, male, 43-year-old, height: 1.83 m, weight: 76.9 kg, maximum oxygen consumption (VO_2_max): 52 ml/kg/min, body fat: 12.6%, systolic and diastolic blood pressure: 120/80 mmHg, blood glucose: 92 mg/dL, total cholesterol (TC): 185 mg/dL, high-sensitivity C-reactive protein (hs-CRP): 0.08 mg/dL, creatine phosphokinase (CPK): 183 U/L, and high-sensitivity troponin T (hs-TnT): 7.1 ng/L. At follow-up, the average of VO_2_max remained at 48.7 ± 1.2 ml/kg/min, left ventricular ejection fraction (LVEF) at 62 ± 2%, LV strain global longitudinal at 19 ± 1%, LV mass index at 83 ± 7 g/m^2^, hs-CRP at 0.07 ± 0.01 mg/L, CPK at 169 ± 36 U/L, hs-TnT at 8.2 ± 1.4 ng/L, and no malignant arrhythmias were observed.

**Conclusion:**

The athlete’s cardiovascular system had adapted to an extremely high volume of consecutive marathons at moderate intensity for one year and remained functioning at normal range. In addition, the athlete set a new world record for most consecutive days to run a marathon, recognized by Guinness World Records.

## Introduction

Endurance performance is determined by maximum oxygen consumption (VO_2_max), running economy, and lactate threshold.^1^ Through regular physical training, it is possible to develop a running economy, especially in long-distance runners.^2^ Impressively, Robison and collaborators reported the physiological profile of a 70-year-old male marathoner who completed a marathon in 2:54:23 on December 15, 2018, breaking the world record time for men over the age of 70 years.^3^

A Belgian athlete ran a marathon per day for 365 days in 2010, accomplishing one of the greatest feats in the history of sport.^4^ However, no clinical evaluation was carried out, and therefore, there is no information about the impact of daily marathons on the cardiovascular system.

A Brazilian amateur marathoner has proposed setting a new world record for consecutive marathons by running 366 marathons in 366 consecutive days. The impact of such a feat on the cardiovascular system remains unknown.

This study aimed to monitor the cardiovascular system regularly to assess any cardiovascular adaptations or maladaptation of the athlete throughout 366 consecutive marathons.

## Material and methods

### Study population

The local committee for the Protection of Human Subjects approved this study (CAAE: 61097822.0.0000.0068). We evaluated a Brazilian male marathoner (H.L.S.F.) who ran 366 consecutive marathons in 366 days (from 28^th^August 2022 to 28^th^August 2023). The participant provided written informed consent.

### Pre-study evaluation

The pre-participation clinical evaluation (PPE) was performed according to the Brazilian Society of Exercise and Sports Medicine.^5^ We performed an anamnesis, clinical examination composed by 12-lead resting electrocardiogram (ECG), maximum cardiopulmonary exercise test (CPET), bioelectrical impedance analysis, and blood test. In addition, the athlete presented an echocardiogram at PPE that was realized three months before our study.

### Follow-up

We periodically repeated all tests. In the third month of follow-up, we also included measures of cardiac structure, left ventricle (LV) and right ventricle (RV) functions by echocardiography (ECHO) and repeated until the end of this study.

In addition, a multi-professional team composed of a dermatologist, endocrinologist, orthopedist, physiotherapist, nutritionist, physical trainer, and psychologist supported the athlete over the period.

### Cardiopulmonary Exercise Test (CPET)

The CPET was performed using the maximum effort on automatic treadmill (Embramed – Model –Atlanta, United States). A computerized respiratory gas analyzer (Vynthus CPX – Pulmonary Function/Cardiopulmonary Exercise Testing Instrument, Hoechberg, Germany) was used to evaluate pulmonary ventilation, maximum oxygen consumption (VO_2_max), and carbon dioxide production (VCO_2_). The variables were measured breath-by-breath. The first anaerobic threshold (AT) was identified by the V-slope technique observed at the first point of dissociation of the VE/VO_2_ curves and the lowest value for partial oxygen pressure at the end of expiration (PetO_2_), before this parameter started to increase progressively. The second ventilatory threshold (respiratory compensation point) was identified by the inflection in the VE/VCO_2_ curves and the maximum value of partial pressure for CO_2_ at the end of expiration (PetCO_2_), before a progressive decrease in this response.^6^ Heart rate (HR) was continuously recorded during the CPET using a 12-lead electrocardiogram and software CardioSoft v6. The CPET was performed at PPE, after three months, and monthly until the end of the study. The athlete ran the marathons in the morning, and CPETs were performed in the afternoon.

### Echocardiogram

The images were collected by the Vivid E9 (GE Healthcare; Oslo, Norway). The athlete underwent 2-dimensional ECHO. The 4-chamber color Doppler and then pulse-wave Doppler were used to assess peak flow velocities across the mitral valve. We assessed parasternal long-axis views, septal and LV posterior wall thickness in diastole, as well as LV chamber dimensions at end-diastole and end-systole. LV volume at end-diastolic and LV volume at end-systolic were assessed by apical 2-and 4-chamber views, which then allowed the estimation of stroke volume and left ventricle ejection fraction by Simpsons biplane method. Specific speckle-tracking software was used to estimate strain in all segments. All the images were collected according to the American Society of Echocardiography and the European Association of Cardiovascular Imaging.^7^ The first echo was performed 3 months after starting the study and periodically evaluated during the follow-up. The athlete ran the marathons in the morning, and echocardiograms were performed in the afternoon, always before the CPET.

### Statistical analysis

Data are periodically presented based on evaluations and reported as mean ± standard deviation (SD). The Statistical Package for Social Science (SPSS) version 23 was used to describe the variables.

## Results

### Pre-study evaluation

At PPE, 43-year-old, height: 1.83 m, weight: 76.9 kg, VO_2_max: 52 ml/kg/min, body fat: 12.6%, systolic and diastolic blood pressure: 120/80 mmHg, blood glucose: 92 mg/dL, total cholesterol: 185 mg/dL, high-sensitivity C-reactive protein (hs-CRP): 0.08 mg/dL, creatine phosphokinase (CPK): 183 U/L, and high-sensitivity troponin T (hs-TnT): 7.1 ng/L.

In the first CPET, we used a specific ramp protocol (protocol: velocity 1) composed of increments of velocity and inclination each minute until the athlete’s exhaustion (protocol details are described in supplementary material). The CPET duration was 10 minutes, the maximum velocity was 8.2 mph, 11.0% of inclination, VO_2_max was 52.0 ml/kg/min, and the respiratory exchange ratio (RER) was 1.11. RER ≥ 1.10 is considered a maximum test.^6^ The first AT was around 6.0 mph, and the HR was 150 bpm. The athlete was instructed to run the marathons at moderate intensity close to the first AT obtained by maximum CPET.

### Follow-up

Clinical measures, body composition, biomarkers, and lipids profile are described in Table 1. These variables showed minimal physiological variations with no abnormal alterations. The resting HR was measured from CPET in the supine position after 2 minutes at baseline. The slight variation of resting HR may be related to pre-test anxiety.

**Table 1:** – Clinical measures, body composition, biomarkers, and lipids profile

Variables	August 2022, Pre-study	2022 November	2023 February	2023 May	2023 July	August 2023, End of Study	Mean - SD	Normal Value or Range
**Clinical**								
SBP (mmHg)	120	110	110	110	120	120	115 ± 5	< 120
DBP (mmHg)	90	80	80	80	80	80	80 ± 5	< 80
Resting HR (bpm)	90	81	96	80	67	77	82 ± 10	< 100
**Body composition**								
Weight (kg)	76.9	74.2	71.4	71.0	71.7	70.5	72.6 ± 2.5	-
Height (m)	1.83	1.83	1.83	1.83	1.83	1.83	1.83	-
BMI (kg/m^2^)	23.0	22.2	21.3	21.2	21.4	21.1	21.7 ± 0.7	18.9 - 24.9
Lean mass (kg)	67.2	65.5	64.2	63.9	64.7	63.6	64.9 ± 1.3	-
Fat mass (kg)	9.7	8.7	7.2	7.1	7.0	6.9	7.8 ± 1.2	-
Fat mass (%)	12.6	11.7	10.1	10.0	9.7	9.8	10.7 ± 1.2	< 20.0
**Biomarkers and Lipids Profile**								
hs-CRP (mg/L)	0.08	0.08	0.06	0.08	0.07	0.06	0.07 ± 0.01	< 0.1
CPK (U/L)	183	184	190	190	183	104	169 ± 36	46 - 171
hs-TnT (ng/L)	7.1	6.0	8.8	10.0	8.0	9.0	8.2 ± 1.4	< 16.8
Creatinine (mg/dL)	1.14	1.01	1.15	1.02	0.99	1.06	1.06 ± 0.07	0.7 - 1.30
Potassium (mmol/L)	4.8	4.3	4.5	4.3	4.7	4.5	4.5 ± 0.2	3.5 - 5.1
Sodium (mmol/L)	137	136	141	138	139	140	139 ± 2	136 - 145
NT-proBNP (pg/mL)	-	-	22.1	24.4	10.8	14.4	17.9 ± 6.4	< 125
TC (mg/dL)	185	180	-	199	191	204	192 ± 10	< 190
LDL (mg/dL)	111	111	-	130	126	132	122 ± 10	< 130
HDL (mg/dL)	53	48	-	52	49	56	52 ± 3	> 40
TG (mg/dL)	99	81	-	75	64	78	79 ± 13	< 150

*HR: heart rate; SBP: systolic blood pressure; DBP: diastolic blood pressure; BMI: body mass index; hs-CRP: high-sensitivity C-reactive protein; CPK: creatine phosphokinase; hs-TnT: high-sensitivity troponin T; NT-ProBNP: N-terminal proBNP; LDL: low density lipoprotein; HDL: high density lipoprotein; TG: triglycerides; TC: total cholesterol.*

The mean HR during the marathons was 140 ± 10 bpm, VO_2_ max remained at 49 ± 1 ml/kg/min, and pulmonary ventilation at 113±4 L/min (Figure 1 A-B). From the CPET performed in January, we changed the protocol velocity 1 to protocol D to avoid the risk of muscle injury during CPET (supplementary material). This alteration could explain a slight drop in VO_2_max and ventilation. However, the athlete reached the maximum HR (HR > 95% predicted by age), and the RER was 1.08 ± 0.04. Moreover, no signs of malignant arrhythmia and cardiovascular disorders were found during CPET, which assured us of the safety of continuing with the marathons. Furthermore, no significant changes between 12-lead ECG in the pre-study and 12-lead ECG at the end of the study were found (Figure 1B-C).

**Figure 1:** – Follow-up of maximum oxygen consumption (VO2max) and pulmonary ventilation by cardiopulmonary exercise test (CPET), (Fig. 1 A-B, respectively). 12-lead resting electrocardiogram (ECG) in the pre-study and ECG at the end of the study (Fig. 1B-C, respectively).

Cardiac structural variables, LV systolic function, RV systolic function, and myocardial work are described in Table 2. These variables showed minimal physiological variations within normal values.

**Table 2:** – Cardiac structural, left ventricular systolic function, right ventricular systolic function, and myocardial work

Variables	October After 3 months	2022 November	2023 March	2023 June	2023 July	August,2023 End of Study	Mean - SD	Normal Value or Range
**Cardiac structures**								
Aortic root (cm)	3.4	3.3	3.4	3.3	3.3	3.4	3.4 ± 0.1	3.0 - 3.7
RA diameter (cm)	3.8	3.5	3.6	3.3	3.4	3.3	3.5 ± 0.1	3.0 - 4.0
RV basal (cm)	3.8	3.8	3.4	4.0	4.0	3.3	3.7 ± 0.3	2.5 - 4.1
RV mid (cm)	3.1	3.1	2.8	3.1	3.3	2.7	3.0 ± 0.2	1.9 - 3.5
Interventricular septum (cm)	0.9	0.8	0.9	0.8	0.9	0.8	0.9 ± 0.1	0.6 - 1.0
LV posterior wall thickness (cm)	0.9	0.8	0.8	0.8	0.8	0.8	0.8 ± 0.1	0.6 - 1.0
LV end-diastolic diameter (cm)	5.4	5.3	5.4	5.3	5.5	5.2	5.4 ± 0.1	4.2 - 5.8
LV end-systolic diameter (cm)	3.5	3.7	3.6	3.4	3.4	3.3	3.5 ± 0.1	2.5 - 4.0
LV diastolic volume (ml)	157	141	153	149	152	149	150 ± 5	80 - 150
LV systolic volume (ml)	62	63	58	59	60	62	61 ± 2	70
LV mass index (g/m^2^)	92	77	87	78	90	76	83 ± 7	49 - 115
LA volume index (ml/m^2^)	18	20	21	16	25	22	20 ± 3	< 34
**Left ventricular systolic function**								
LV ejection fraction (%)	60	62	62	61	60	59	61± 1	> 52
LV global longitudinal strain (%)	16	18	19	19	20	19	19 ± 2	≥ 18
**Right ventricular systolic function**								
TAPSE (mm)	21	24	24	22	24	25	23 ± 2	> 17
S’ (cm/s)	14	13	14	14	15	19	15 ± 2	> 9.5
FAC (%)	49	46	53	51	46	50	49 ± 3	> 35
RV strain (%)	21	24	23	25	23	22	23 ± 1	> 20
Myocardial work								
GWE (%)	95	-	92	92	97	94	94 ± 2	> 96
GWW (mmHg)	84	-	182	154	49	106	115 ± 53	73 - 87
GWI (mmHg%)	1576	-	1953	1688	2148	2025	1878 ± 238	1907 - 2113
GCW (mmHg%)	1680	-	2194	1987	2224	2209	2059 ± 233	2186 - 2369

*RA: right ventricle; RV: right ventricle; LV: left ventricle; LA: left atrium; TAPSE: tricuspid annular plane systolic excursion; S’: tricuspid lateral annular systolic velocity wave; FAC: fractional area change; GWE: global work efficiency; GWW: global work wasted; GWI: global work index; GCW: global constructive work.*

In addition, the athlete had been involved in a muscle strengthening program based on weight training, mobility, flexibility, and stretching, twice a week, 2 to 3 sets to each muscle group with 15 repetitions at 70% of your maximum capacity during the marathons. The average daily calorie consumption was 4.716 ± 806 kilocalories (composed by carbohydrate 68 ± 6 %, lipids 17 ± 5%, and protein 15 ± 4%). Psychological work was also performed by the athlete, composed of 102 therapy sessions that assessed personal, motivational, resilience, ability to deal with frustration, family involvement, routine division, and adaptation over the period. In January 2023, the athlete was diagnosed with pubalgia and needed physical therapy treatment that remained until August 2023. The athlete documented all the marathons and distances using two smart watches simultaneously and used 27 pairs of running shoes. The average marathon time was 5 hours, 8 minutes, and 28 seconds; the fastest was 3 hours, 54 minutes, and 48 seconds; and the slowest was 10 hours, 21 minutes, and 6 seconds.

## Discussion

To the best of our knowledge, this is the first study to monitor the cardiovascular system regularly in a very comprehensive manner in a male marathoner who ran 366 daily consecutive marathons. We hypothesized that the athlete would undergo physiological cardiovascular adaptations or maladaptations due to the high volume of running in one year. Interestingly, the athlete presented minimal physiological variations with no abnormal cardiovascular changes in one year. In addition, the athlete set a new world record of running 366 consecutive marathons in 366 days, recognized by Guinness World Records.^8^

First, we performed PPE to identify the presence of unknown cardiovascular diseases that could be incompatible with this study. The athlete had no family history of sudden cardiac death or hypertrophic cardiomyopathy and showed a great cardiovascular, respiratory, hemodynamic, and physical response during CPET.^9^ The 12-lead resting ECG, blood test, and blood pressure were within the normal limits. Furthermore, the athlete presented an echocardiogram with normal values that was realized three months before our study.

Exercise training is a powerful trigger to promote structural and functional remodeling of the heart.^10^ However, there is increasing recognition of the impact of prolonged exercise training on cardiac remodeling, which may eventually mimic certain pathological conditions.^11^ During the follow-up, we periodically monitored the athlete to guarantee his safety, and if eventually the athlete presented any pathological condition that could harmfully progress with potential risk to his life, the study would have been interrupted. Interestingly, the athlete presented minimal physiological variations in cardiac structure, LV function, and RV function, with no signs of overtraining or altered inflammation over the period.^12^

Controversially, exercise training may be associated with an increased risk of ventricular and atrial arrhythmias.^13^ Moreover, exercise intensity but not volume may be associated with increased coronary calcification in athletes.^14^ In our study, the athlete was instructed to run the marathons at moderate intensity (around first AT), and no signs of altered ECG suggesting ischemia and malignant arrhythmias were observed during CEPT.

Studies to understand physiological responses in champion athletes with performance records have been carried out for several years.^1^ VO_2_max is a marker of cardiac respiratory and circulatory response, which is one of the main factors that contribute to endurance performance.^1^ Furthermore, steady-state oxygen consumption is often referred to as a running economy. The 70-year-old male marathoner, who ran a marathon in less than 3 hours in 2018, breaking the world record for men over 70, performed a single CPET and demonstrated a VO_2_max of approximately 46.9 ml/kg/min. This value was exceptional for his age, as the average VO_2_max for an age-matched male is around 26 ml/kg/min.^3^ In contrast, no clinical evaluation was performed on the Belgian athlete who ran 365 consecutive marathons, highlighting the uniqueness and significance of our findings.

In our study, the CPETs were performed in the afternoon, after the athlete had completed a marathon earlier in the day. This may have influenced performance metrics, including VO_2_max, ventilatory thresholds, and cardiac responses, potentially underestimating the athlete’s true maximal capacity. However, the athlete showed an optimal VO_2_max and sustained adaptations in physical capacity throughout the 12 months (Central Illustration).

### Clinical implication

These results highlight the importance of a careful cardiovascular assessment in athletes undergoing endurance exercise and provide unique insight into the relationship between high-volume, intensity marathons and cardiovascular adaptations.

### Limitations

We recognize some limitations in our study. From the CPET performed in January until August, we adjusted the protocol velocity to reduce the risk of muscle injury during CPET. This alteration may explain a slight drop in VO_2_max and ventilation, which could limit the scientific utility of VO_2_max as a follow-up variable. However, VO_2_max remained around 49 ml/kg/min, suggesting a sustained functional capacity. The Echo was not performed in the pre-evaluation study because the athlete presented an echocardiogram with normal values at PPE that was realized three months before our study. However, during the follow-up, the parameters were within the normal limits. This study relates to one male marathoner, and these results should be interpreted with caution when extrapolating to a broader population.

## Conclusion

In conclusion, the athlete’s cardiovascular system had adapted to an extremely high volume of consecutive marathons and remained functioning at a normal range. In addition, the athlete set a new world record of running 366 consecutive marathons in 366 days, recognized by Guinness World Records.
